# Adopt, adapt, or abandon technology-supported person-centred care initiatives: healthcare providers’ beliefs matter

**DOI:** 10.1186/s12913-021-06262-1

**Published:** 2021-03-17

**Authors:** Kari Dyb, Gro Rosvold Berntsen, Lisbeth Kvam

**Affiliations:** 1 Norwegian Centre for E-health Research, University Hospital of North Norway, P.O. Box 35, N-9038 Tromsø, Norway; 2 Department of Mental Health, Faculty of Medicine and Health Sciences, Trondheim, Norway

**Keywords:** Technology support, E-health, Patient-centred care, NASSS framework, Healthcare providers

## Abstract

**Background:**

Technology support and person-centred care are the new mantra for healthcare programmes in Western societies. While few argue with the overarching philosophy of person-centred care or the potential of information technologies, there is less agreement on how to make them a reality in everyday clinical practice. In this paper, we investigate how individual healthcare providers at four innovation arenas in Scandinavia experienced the implementation of technology-supported person-centred care for people with long-term care needs by using the new analytical framework nonadoption, abandonment, and challenges to the scale-up, spread, and sustainability (NASSS) of health and care technologies. We also discuss the usability and sensitivity of the NASSS framework for those seeking to plan, implement, and evaluate technology-supported healthcare programmes.

This study is part of an interdisciplinary research and development project called Patients and Professionals in Partnership (2016–2020). It originates at one of ten work packages in this project.

**Method:**

The main data consist of ethnographic field observations at the four innovation arenas and 29 interviews with involved healthcare providers. To ensure continuous updates and status on work in the four innovation arenas, we have also participated in a total of six annual network meetings arranged by the project.

**Results:**

While the NASSS framework is very useful for identifying and communicating challenges with the adoption and spread of technology-supported person-centred care initiatives, we found it less sensitive towards capturing the dedication, enthusiasm, and passion for care transformation that we found among the healthcare providers in our study. When it comes to technology-supported person-centred care, the point of no return has passed for the involved healthcare providers. To them, it is already a definite part of the future of healthcare services. How to overcome barriers and obstacles is pragmatically approached.

**Conclusion:**

Increased knowledge about healthcare providers and their visions as potential assets for care transformation might be critical for those seeking to plan, implement, and evaluate technology-supported healthcare programmes.

## Background

Governments across the Western world, together with private enterprises, healthcare providers and patient organisations, are emphasising the need for healthcare to be more explicitly centred on the needs of the individual patient, prioritising the philosophy and practice of person-centred care (PCC) as the core of new and effective models of care delivery [1–5]. PCC is acknowledged as a key component of quality healthcare for chronically ill patients [6, 7]. The hallmark of PCC is partnerships between patients and healthcare providers to enhance patients’ active, day-to-day involvement in their health [8]. Such interactions do not require face-to-face visits but may be ensured by using computer technologies [9, 10]. In fact, health information technologies may be important facilitators for PCC [11, 12]. In this paper, we investigate how healthcare providers experienced the implementation of technology-supported PCC for chronically ill patients by using the new analytical framework nonadoption, abandonment, and challenges to the scale-up, spread, and sustainability (NASSS) of health and care technologies [13].

The rationale for implementing technology-supported PCC relates to the general development trends of most Western societies, such as demographic changes, growing social and cultural inequalities, and greater health expectations. Governments expect an increase in both the absolute number and proportion of elderly individuals in the population, many with chronic and complex medical conditions, and have invested in technology-supported solutions to meet these societal changes [14, 15]. In Nordic countries, Danish health authorities state that the only option is increased digital collaboration [16] while Norwegian authorities refer to digitalisation as a means of increasing patient involvement and democratisation [5].

While few would argue with the overarching philosophy of PCC, nor with the potential of information technologies, there is less agreement on how to make technology-supported PCC a reality in everyday clinical practice. Researchers argue that there is a significant gap between the enthusiasm, high hopes, and expectations of policy makers, managers, and IT developers and the challenges of technology implementation in actual practices [17], and they point to the need for new studies of what happens in clinical practices when governments try to modernise healthcare services with the help of information technology. Studies can better inform decisions about health policies, programmes, and practices [18] and aid those seeking to design and implement such initiatives to identify and address key challenges [13, 19, 20]. There is both a need to understand how complex practices are made workable and integrated in context-dependant ways [21] and to theorise on challenges and failures to adopt or normalise technology-supported programmes [19]. Even now, seemingly well-functioning technology trials tend to fail final implementation into daily practices, and the failure of technology implementation is often not due to individuals alone [22]. Studies must therefore look into the dynamic interaction between health personnel, patients, the technology in use, team functioning, and the economic, governance, and regulatory factors [22, 23]. All these factors and more may be facilitators or barriers in implementation processes.

In this paper, we will contribute to the implementation debate by empirically exploring, comparing, and theorising the experiences of individual healthcare providers in four arenas where digital technologies have been implemented to support PCC for people with long-term needs. The rationale for focussing on the experiences of the involved healthcare providers is that while policymakers, managers, and IT developers may invest in technology-supported PCC, it is up to health service employees to put it into practice [24], and knowledge about staff acceptance is still limited [25]. We do this by applying the new NASSS framework [13]. We will also discuss the usability and sensitivity of the NASSS framework for those seeking to plan, implement, and evaluate technology-supported healthcare programmes.

### Study context

There is little disagreement in Nordic countries that the public should pay for the most important services in education, health, and social services [26]. Healthcare services build on a classical Nordic welfare model, combining financing and provision of universally accessible services, mainly within the public sector [27]. The countries rank high on the OECD list of health spending per capita [28], their governments invest heavily in healthcare technologies [4, 5], and the population is generally well educated with high levels of internet access [29]. Consequently, Nordic countries are particularly interesting for studying technology-supported PCC initiatives.

This study is part of a research and development project called Patients and Professionals in Partnership (3P) [30]. 3P was funded from 2015 to 2020 through a grant from a cross-regional health research fund owned by the four Norwegian Regional Hospital Trusts (Helseforsk). The purpose of 3P is to answer the urgent call from health institutions, healthcare providers, and health authorities to radically redesign care delivery for patients with long-term and complex needs. Based on the principles of the chronic care model [31–33], 3P includes four Nordic initiatives that have implemented PCC models, all of which take advantage of new technologies and radical organisational redesign to transform classical, profession-centric healthcare systems towards citizen-centric health delivery systems.

Prior to the 3P project, the four initiatives, which we refer to as innovation arenas, were autonomous PCC initiatives with independent funding and management, following project logic with a launch date for the innovation and an ending date of the project. The arenas are in different healthcare trusts, three in Norway and one in Denmark. All share the vision of using innovative technology to develop healthcare that is truly citizen-centred; is coordinated, proactive, and planned; has one point of contact; uses interdisciplinary teams; and is a learning care system. The aim of 3P is to validate and verify the prerequisites that support a whole healthcare system redesign towards the quadruple aim of improved outcomes, improved care experiences, improved professional experiences, and reduced costs [34]. This paper originates in one of ten work packages in the project, the implementation study, and is led by social scientists.

### Theoretical approach

The NASSS framework considers seven domains: the illness/condition, the technology, the value proposition, the adopter system, the organisations, the wider context and the interaction between them. The complexity of each component is essential to predict and evaluate the success of technology-supported healthcare programmes [13]. The framework encourages complex thinking about technological innovations in healthcare, aiming to generate a rich narrative of events unfolding in a real-world setting. It illustrates a variety of challenges across all domains, each classified as either simple (straightforward and predictable with few components), complicated (multiple interacting components or issues), or complex (dynamic, unpredictable, not easily disaggregated into constituent components). It demonstrates how technology programmes characterised by complicatedness prove difficult but not impossible to implement, while those characterised by complexity in multiple NASSS domains rarely, if ever, become mainstreamed.

This is based on a narrative systematic review and empirical work. It was first published in 2017 by Trisha Greenhalgh and colleagues [13]. To our knowledge, current publications cover the development and extension of the NASSS framework [35–37], empirical applications of the NASSS framework [12, 38–45], reviews and synthesis guided by NASSS [46], and a few research protocols based on the framework [47–49].

Using theoretical frameworks can help theorise on, predict, and evaluate the success of interventions and processes in healthcare [12], and we use them to capture the heterogeneity of social interactions in a naturalistic rather than experimental way [17]. In this paper, we use NASSS as a theoretical framework to explore the embedding and integration of four technology-supported PCC initiatives from the perspectives of healthcare providers; concurrently, we use the empirical studies to inform theory development by exploring the usability of the theoretical constructs.

Additionally, the 3P project consists of ten separate work packages and has practised ongoing interaction with the project management team, key healthcare providers, and interdisciplinary researcher group. This paper report from the implementation study, and two of the authors are sociologists. As sociologists facilitating interdisciplinary discussions about implementation science, we have explored the applicability and usefulness of NASSS as a framework for engagement and knowledge sharing within an interdisciplinary audience.

## Method

The study design is explorative, based on an understanding that there is almost inevitably a crucial gap between what is possible to deliver technically and the nuanced, flexible, and often unpredictable nature of human activity [17].

### Data collection

In the period between 2016 and 2018, we visited each innovation arena at least once. During these field visits, the first and last author stayed in the local towns for three to 5 days to obtain in-depth knowledge about the innovation arena and its wider context. We observed the healthcare providers in naturalistic settings and explored their working environment, the technologies present, the buildings, the rooms, and the room layout. We also investigated patient-provider consultations and interactions between professionals, departments, institutions, and levels of care. In addition, we looked at the local communities and municipalities, the towns, the local infrastructure, and the surrounding geographies. The description of the innovation arenas is built on the field visits.

The main data consist of interviews with involved healthcare providers. We used the broad meaning of the term and conducted 29 interviews with nurses, physiotherapists, occupational therapists, nutritionists, doctors, logisticians, IT personnel, clinical and administrative managers, and local policy makers to gather in-depth knowledge from the diverse ranges of professions involved in providing technology-supported PCC in the four arenas. The interview guide was semi-structured and informed by both the preliminary observations and the NASSS framework. Most interviews were individual, but a few were group interviews with two or more participants. A total of 36 heath care providers were interviewed. Most participants were heavily involved in one of the innovation arenas and familiar with the 3P project. To some extent, they were also familiar with our research. We started the interviews by asking them to tell us their own stories about the technology-supported PCC initiative and stressed that they should take their time, that there were no right or wrong answers. Most interviews were between 60 and 90 min long and took place primarily at the informants’ workplaces, typically a private office or a meeting room at a hospital or municipal care centre. All participants signed an informed consent form.

Throughout the span of the 3P project (2016–2020), we participated in six of the seven network meetings arranged by the project. The four arenas arranged one or two meetings each, while the project management was responsible for the kick-off meeting and the end-of-project symposium, scheduled for December 2020. Each meeting lasted for two to 3 days and involved around 40 participants, consisting of the project managers and key healthcare providers at the four innovation arenas, local actors involved in similar technology-supported initiatives, local and national policy makers, and the 3P researchers. Some network meetings included site visits. All meetings included lectures, groupwork, and social gatherings. In this way, the network meetings ensured continuous updates and status on work at the four innovation arenas, as well as feedback on the preliminary research findings. All methods were carried out in accordance with relevant guidelines and regulations.

### Analysis

The first and the last author conducted the field work. During the field visits, we discussed the day’s work and compared field notes, on-site observations, and interview experiences at night. When returning from the field work, we had all the recorded interviews transcribed, and we coded the field notes and the interviews separately. Then we met to discuss the emerging findings via systematic reading and deductive coding informed by the NASSS framework. We used the network meetings to support and contest the emergent findings.

After completing data coding for all innovation arenas, we met again to discuss and compare the findings and the applicability of the seven domains across the arenas. Towards the end of the analytical work, we debated the usefulness and sensitivity of the NASSS framework on our existing empirical material.

## Results and discussion

In Norway and Denmark, equal access to high-quality health and care services is a legal right for all citizens. Nordic health and care services are prevailingly public amenities, tax funded, and with low patient imbursement [50]. The services are organised into primary and secondary care. In Norway, nursing homes, general practitioner (GP) offices, and home care services are part of primary care, while hospitals fall under secondary care. In Denmark, primary care services consist primarily of nursing homes and homecare, while five independent healthcare trusts are responsible for the hospitals and GP services in the country.

### Innovation arena 1

Arena 1 was in Denmark and in a mature phase of the innovation. It provided technology-supported PCC to approximately 90 patients with chronic obstructive pulmonary disease (COPD) living in a municipality close to the capital. COPD is a lung condition characterised by breathing difficulties due to damage to the air sacs in the lungs (emphysema) and long-term inflammation of the airways (chronic bronchitis). Prior to the innovation, most COPD patients lived at home and received GP services when they had clinical deterioration. The innovation consisted of a specific care model developed by the key healthcare providers. The aim of the model was to enable the highest possible degree of independent, individualised, and active living for patients with chronic conditions. The model provided one point of contact for the patients. It consists of six categories in which the patient, which the model refers to as a citizen, is monitored and assisted virtually or physically, either at home or within the health services depending on their current health condition. Each of the six categories represents a defined level of care, and patients move between levels depending on their current conditions. Level 1 is the optimal level for patients, representing mobility and individualised daily living under full self-control. At this level, patients are virtually supported by the response and coordination centre, which is their one point of contact and is responsible for monitoring each patient for vital and COPD indicators. If a patient’s condition worsens, they can activate one point of contact to obtain virtual assistance instantly from a nurse or a doctor (e-doctor) (level 2). If virtual assistance is not enough, the patient is moved to level 3. At this level, a professional (typically a nurse) will come to the patient’s home and perform different kinds of examinations, including ECG and blood samples, and videoconference with the e-doctor. If the e-doctor decides to intensify the treatment, the patient is moved to level 4, where they are ‘outmitted’. This means that the patient is still at home but receiving intensive online synchronous and asynchronous monitoring and treatment. If this is not enough, the e-doctor admits the patient either to the local health clinic (level 5) or to the hospital (level 6). The arena was organised and staffed as an integrated part of the municipal health and care services, and employed two dedicated e-doctors, who provided care exclusively through electronic tools, in addition to pre-existing municipal staff.

### Innovation arena 2

Arena 2 was on the west coast of Norway and in a mature phase of the innovation. It offered technology-supported PCC for COPD patients in their homes for the first 14 days after hospital discharge. Prior to the innovation, the COPD patients had to travel to the hospital for planned follow-ups. The innovation was a nurse-led telemedicine service placed in a local hospital, delivering PCC to about 50 patients a year through remote monitoring of vital COPD indicators and daily videoconferences with COPD patients at home, focusing on self-management support. The virtual care involved nursing, physiotherapy, occupational therapy, and nutritionist care. The telemedicine service was staffed with two full-time nurses, while the therapists provided virtual care to COPD patients living in the local town and in neighbouring municipalities as part of their regular duties at the hospital. The online care was primarily organised by the nurses and therapists, but patients could book or rebook appointments according to their preferences.

### Innovation arena 3

This arena was in the southern part of Norway. It offered technology-supported PCC care to COPD patients living at home. The telemedicine innovation was tailored to COPD patients in acute situations or with worsening chronic conditions who are living in municipalities within a specific geographic area. The redesign of care involved the establishment of an interdisciplinary team from primary and secondary care with the COPD patient as the core team member.

At the time of our research, the innovation arena was in a start-up phase and had recruited five patients, all of whom received municipal healthcare services prior to the innovation. Two specially trained nurses at a telemedicine centre located in a municipal nursing home ran the service as part of the home care services provided by the nursing home. The nurses could monitor vital COPD indicators from the centre and communicate virtually with the patients, who used personal tablets as a means of communication from their homes. The communication was primarily synchronised and initiated by the nurses; however, both patients and healthcare providers could initiate virtual contact.

Like arena 2, arena 3 originated from a previous technology-supported PCC project. The innovation was a result of close collaboration between the local university, several of the municipalities in the region, one nursing home, and the two hospitals in the region.

### Innovation arena 4

This arena was in North Norway. It was in a mature phase of the innovation and employed approximately 15–20 staff and included about 400 patients annually. It was a collaboration between a university hospital and the healthcare services in a few nearby municipalities, and targeted patients receiving both hospital and municipal services. It emphasised the need for interdisciplinary teams and collaboration and included nurses, physiotherapists, occupational therapists, pharmacists, nutritionists, and doctors from municipal and hospital services working as a unified team with the patient, continuously placing emphasis on what is important for the individual patient. The target group was elderly and frail patients with multiple or chronic conditions at risk of acute (re)hospitalisation. The redesign was to coordinate the care for these patients and, above all, the transference between primary and secondary services. The innovation was patient centredness and holistic, proactive care, and the services were mobile. Healthcare was provided in patients’ homes, municipal healthcare institutions, and hospitals. To support PCC, the healthcare providers had access to both municipal and hospital electronic health records (EHRs), and the arena applied an explorative approach towards patient-facing technologies.

### Domain 1 – the condition

The first NASSS domain addresses the clinical condition, impending comorbidities, and sociocultural aspects of the condition, exploring whether patients are appropriate candidates for the use of this technology. It recognises that only a fraction of potential end users are assessed by their clinicians as suitable for the technology and that the condition is often considered clinically high risk, unpredictable, or atypical (e.g., complicated by comorbidities or sociocultural factors, especially cognitive or health literacy considerations).

In our study, three of the innovation arenas targeted patients with COPD, while the fourth provided care for elderly and fragile patients with comorbidities. Across the arenas, healthcare providers agreed that COPD patients with both simple and complex comorbidities were well suited for technology supported PPC. They argued that the disease often leads to anxiety, insecurity, loss of appetite, and inactivity and that technology use was helpful through easy and frequent accessibility and visual support from healthcare providers. One asserted that ‘it is not morally sound not to take advantage of the possibilities in technology-supported healthcare’ (Informant 1). This informant argued that increased knowledge about the complexity of a condition and its interdependencies increases the significance of remote monitoring, as distance monitoring provides better assessment of when hospitalisation is necessary, when small adjustments in treatment were sufficient, or when just talking to the patient was enough.

Another said, ‘I think it is best suited for patients in early stages of COPD; however, very ill patients need it even more’ (Informant 2). This informant continued by emphasising, ‘each COPD patient, but particularly very sick patients, needs to feel safe and secure and get quick responses from professionals (informant 2), indicating that technology support could provide safety and security to very sick patients with complex comorbidities.

Contrary to the COPD arenas, in the innovation arena where the condition was multimorbidity and frailty, the healthcare providers problematised technology support for patients with complex conditions. Here, they had tested different tools for digitised patient-provider communication but had not implemented a specific technology yet. Several argued that the frailest patients were too frail to manage this technology’s use.

According to NASSS, complex conditions and complexity in an underlying condition are associated with non-adoption, abandonment, or limited usefulness of the technologies. Conditions like dementia or multi-morbidity often make a patient unable or unwilling to use the supplied technologies [39]. In our study, the healthcare providers described COPD on a continuum from easy to severe, and they acknowledged that patients often had additional challenges, such as anxiety, malnutrition, isolation, and depression. However, they did not problematise COPD as too complex for technology support at any level of severity. Frailty, on the other hand, was described as too complex for technology support. It is interesting to note that the most experienced technology support users were the least concerned about the complexity of the condition.

### Domain 2–the technology or technologies

This domain addresses questions about the material and technical features of the technology, the knowledge generated or made visible by technology, the knowledge and support needed to use the technology, and the sustainability and supply models.

The technologies used in the three COPD arenas can be defined as freestanding telemedicine solutions, involving iPads and monitoring devices in patients’ homes and videoconference systems at nurse-led call centres. The technologies opened a virtual dialogue between the patients and providers, including medical, nursing, physiotherapy, nutritional, and occupational therapy tasks. They did so by sending biometric data from the patients’ homes to the healthcare system and sending advice and instructions or reminders from the healthcare system back to the patients. All the COPD arenas used locally developed software, which allowed easy access to technical expertise and potentially critical technical issues to be resolved in an ongoing way.

We witnessed complaints or dissatisfaction with the technology in only one arena. Here, healthcare providers complained about the quality of the videoconferencing technology. One said, ‘Sometimes you only see part of the patient’s head, as the patient doesn’t know how to place the iPad properly. There is a range of technical issues. The picture is not clear, or the patient’s face appears green. We can’t trust it to be accurate’ (Informant 3). In another arena, nurses could control the patient-facing iPads remotely from the call centre, and they emphasised the usefulness of this functionality. One said, ‘If the patient is not able to touch the screen of the iPad and the green telephone icon, we are able to oversteer it, so the patients only need to sit down in front of the iPad’ (Informant 4). Here, they did not complain about any technical challenges. Rather, they described the technology as accurate, trustworthy, and sufficient for shared decision-making and high-quality care. Close monitoring of changing symptoms made medication and adjustment of medication accurate, and video communication made it easy to assess patient needs, including determining if the patient was okay or if they needed more intensive care, like hospitalisation.

According to healthcare providers, COPD often includes fluctuating energy and respiration levels. These conditions were moderated by the technology as it allowed increased self-determination and minimised stress related to travels to doctor’s appointments. It also minimised stress related to feelings of guilt and shame over having COPD, as COPD is a stigmatised condition, even regarded as self-inflicted, something that could make patients reluctant to see their doctors. Other healthcare providers highlighted the advantage of seeing and monitoring patients in their everyday environments and not in an institutional context. For example, one therapist said,

Many patients sink back in their chair; this makes it more difficult to breathe. When we can observe them in their own chair and own living room while they are actually doing normal and everyday things, it is easier to give accurate instructions, to help them make adjustments for a better sitting position for breathing, or to perform exercises to ease breathing adjusted to their actual environment. (Informant 4)

The technology innovation in arena 4, which was targeting elderly and frail patients with multiple or chronic conditions, was a complex procedure of documenting in two different EHR systems, one for primary care and one for secondary care. At the time of this research, they had not yet implemented any specific technology for remote monitoring. The use of iPads or similar technologies for remote care was optional. The aim was to use commercial, off-the-shelf technologies, and at the time, just a few staff members had tested iPads for remote communication, and only for staff-to-staff interaction. The iPads had not yet been used for patient-provider interaction.

In general, the healthcare providers were positive about technology and remote care if it was beneficial for PCC. A few described video communications as useful between the nurses visiting the patients’ homes and the occupational or physiotherapists at the workplace. Such virtual visits provided the therapists with important knowledge, for example, on potential obstacles, like thresholds, steep stairways, or narrow bathrooms that would need readjustment, without travelling to the patients’ homes.

The NASSS framework equalises technology complexity with non-adoption or limited use [13, 36]. Our data reveal some variation between the arenas when it came to technology satisfaction. However, it is our understanding that this variation reflects stages of the implementation processes rather than differentiated levels of technical complexity. The arenas in the late or final phases had the most adjusted technologies and the most experienced users. Here, the healthcare providers had few technical challenges, and none complained about poor quality or complex user interfaces; they had also established better routines for technology deficits than the other arenas. In the arenas in the late phases of implementation, the technologies were straightforward, predictable, and simple to use.

Nevertheless, in line with Tolf et al. (2020) [44], our study also demonstrates how technology assessments are not solely related to the simplicity, functionality, and accuracy of the technology itself; they also enhance assessment of the care provided through technology support. Strongly motivated by providing proper PCC, the healthcare providers in arena 4 had adopted what they described as a cumbersome and time-consuming procedure of documenting in two different EHR systems.

### Domain 3 – value proposition

The third NASSS domain considers the value of the innovation and for whom it generates value. It questions whether a new technology is worth developing in the first place and includes both the upstream values that follow the supply-side logic of financial markets and investment decisions and the downstream values that follow the demand-side logic of health technology appraisal, reimbursement, and value for patients.

In our study, some project leaders and managers expressed concern about the upstream value proposition of the innovation. One said, ‘It [the current initiative] is not the only reason to carry out this project. We ought to set up a technical platform that can be of use for initiatives beyond this project to justify the resources’ (Informant 5). This project leader saw the innovation arena as part of a greater whole and acknowledged the need to make the technology profitable for investors to achieve adoption, spread, and scale-up. Another highlighted the importance of developing economically sustainable technology: ‘The technology itself must become economically sustainable. Today, it is very expensive and time consuming to update and maintain the technology. This must be sorted out, otherwise the initiative will fail’ (Informant 6). These two observations reflect how some project leaders saw the upstream value proposition of technology development as critical, emphasising how new technology must possess qualities that allow for commercial trade after the end of the project period. Despite the voiced concerns, only one of the innovation arenas indicated that it had developed an explicit business model, including a planned distribution of reimbursements.

A couple of the healthcare providers from primary care problematised the potential of increased cost with PCC. They referred to situations where COPD patients were assigned to technology support at home, after hospitalisation. A few of these patients had not received municipal homecare services previously; consequently, it appeared that the technology-supported PCC increased rather than decreased costs, at least in the short term. At the time of this research, there was no way of knowing if the innovation enrolled new patients and thereby increased costs or if these patients had been enrolled in homecare services anyway due to the increased severity of their COPD condition.

While a few managers were concerned with the importance of economically sustainable technology, healthcare providers in general were engaged in downstream values. Some even conveyed scepticism concerning the ability to render easily recognised economic gains and criticised the ubiquitous focus on business models in healthcare. One asked rhetorically, ‘Which part of the healthcare services should benefit economically from new e-health innovations?’ (Informant 7). Another stated, ‘When you present at conferences, the first question asked is “What is your business model?”’ (Informant 8), dejectedly explaining why developing a sustainable business model was challenging. ‘Today, municipalities do not get any reimbursement from using e-health technology. The general practitioner can use interdisciplinary reimbursement and the hospitals might save money from reduced stays in hospitals; however, to let a nurse do the job of a doctor, that yields no economic benefit’ (Informant 8).

Across the four arenas, the true value proposition was improved quality of life for patients. The healthcare providers also described technology-supported PCC work as fulfilling and enjoyable for themselves as professionals: ‘I observe how the use of the technology contributes to better lives for the patients, which is very fulfilling for me, too’ (Informant 9). In addition, they described technology-supported PCC as a useful approach to meet the so-called ‘silver tsunami’, indicating an increased number of elderly individuals with chronic conditions.

It is our understanding that there was a mismatch between the upstream and the downstream value proposition at all four arenas, materialised through emphasising improved quality of life for the patients as the true value proposition of the innovations rather than business models and specific plans for commercial spread of the technology. According to NASSS, complexity in the value proposition is associated with limited adoption; in our study, this complexity or mismatch might also have strengthened the healthcare providers’ belief in technology-supported PCC.

### Domain 4 - the adopter system (staff)

The fourth NASSS domain is about adoption and continued use of the technology by the patients, their next of kin, and the staff. Since our study is about healthcare providers’ experiences, we have explored the staff’s adoption or abandonment of technology-supported PCC, including staff engagement with the vision, whether they used the technologies or not, and whether they had concerns about threats to their professional role, scope of practice, or identity.

Most of the staff—in fact, all the informants in our study—were positive towards technology-supported PCC. All talked about PCC as something exclusively positive. In one innovation arena, the key initiators were nicknamed ‘the Three Musketeers’, illustrating their dedication towards the vision and each other. In another arena, the opponents, fighting for the same grants, had sarcastically labelled the initiative ‘the castle in the air’ to highlight the idealistic dimension of the innovation. The healthcare providers embraced technology-supported PCC to such an extent that the authors decided to reread the interview data, explicitly looking for blind spots, nuances, or discrepancies in their positive attitudes. Their passion for care transformation also differs from Kadesjö Banck and Bernhardsson’s study, using the NASSS framework to explore therapists’ and managers’ experiences during a pilot implementation of internet-delivered cognitive behavioural therapy for insomnia in psychiatric healthcare in Sweden [39]. They report that the key barrier for adoption concerned the adopters of new technology, particularly the therapists and their competing demands leading to low prioritisation of the technological innovation.

However, the reread confirmed our initial analysis. Furthermore, it made us aware of the distinction between how the involved staff described their own feelings towards technology-supported PCC and how they described colleagues’ and others’ attitudes towards the same phenomenon. The latter group was portrayed as far less convinced than the former. To convince others about the significance of PCC compared to traditional care was described as a continuous struggle. It was also a distinction between the healthcare providers committed to clinical work and those with managerial duties. While the first group, to a larger extent, referred to its own experiences and aims within the innovation arena, the second group was more visionary, referring to ‘the acknowledged need for care transformation’ and ‘the bright future of technology-supported PCC’. To them, adoption was complex and unpredictable by nature. The current innovations were portrayed as pieces of the puzzle of healthcare services for the future.

Although there was an overall positive attitude among staff, it is useful to differentiate between the healthcare providers’ visions for PCC and their experiences with technology support. In the arenas in which the technologies were up and running, the staff were exclusively positive about technology support, describing it as physically and emotionally beneficial for the patients. Remote monitoring ensured accurate clinical care, and one point of contact ensured safety and security. A few even argued that proper PCC involved technology support. The staff at the arenas for which technology support was less developed were also unambiguously positive towards PCC; however, they were a bit more ambiguous towards technology support. While some described it as unethical not to use technology, others emphasised that technology needed to be handled with care. It could and should not replace face-to-face care.

Some healthcare providers described technology-supported PCC as an entirely new way of approaching the patient. One said, ‘I had to learn how to do this. I asked my colleagues a lot in the beginning’ (Informant 10). Being involved in technology-supported PCC projects did not only mean learning new tasks; it also meant that established routines and professional roles were up for revision. One nurse said, ‘The best thing is that we have much easier access to the e-doctors than we have to regular doctors; the e-doctors are at hand when we need them’ (Informant 11). Others emphasised the professional advantages of working interdisciplinarily. One said, ‘I like working as a team’ (Informant 12). However, there were challenges in all the arenas, as one informant emphasised:

To work in a patient-centred way, we need truly interdisciplinary teams. We need competent nurses and doctors who can see the whole picture. Health issues, social matters, organisational aspects, and so on. Doctors are often not interested in telehealth solutions. PCC and telehealth require a new approach. You must think like a health minister to see the whole patient and his or her situation; most doctors are not trained to think holistically. (Informant 8)

In addition to non-adoption and abandonment due to usability challenges by staff, the NASSS framework refers to staff concerns about threats to their scope of practice, or to the safety and welfare of the patients, and even fear of job loss. In our study, the staff favoured the vision of technology-supported PCC. Some had even sacrificed full-time positions or worked without payment at times to fulfil the vision. Most argued that technology-supported PCC would benefit the patient, themselves as professionals, the healthcare system, and society as a whole. It is our understanding that despite setbacks and struggles with adoption and continued use, the involved staff’s commitment to the innovations was solid.

### Domain 5 - the organisation(s)

The fifth NASSS domain refers to the capability and readiness of organisations for innovations. It addresses the organisations’ capacity to embrace any service-level innovation, the readiness for a specific technology, and the interdependencies between organisations.

In all four innovation arenas, the involved organisations had extended expertise in managing and implementing projects. Some had dedicated research and development departments, and two arenas were led by professional project leaders employed in such departments. Consequently, it seemed like the involved organisations were capable and ready for the innovations. However, the healthcare providers experienced that while the organisations were ready for innovation projects, they were not ready for long-term changes and the transformation of day-to-day practices: ‘It [the innovation] worked very well as a project. When it needed to be adopted as part of daily practice, on the other hand, it became very difficult’ (Informant 12).

According to the healthcare providers, challenges with transitioning from a project to normalised care were to a large extent related to a lack of funding. While funding was taken care of and agreed on during the project phase, the demand for cost-benefit analysis increased after this phase. All expressed concern about tight budgets and short-term funding. Some described disputes about resources within the organisation, and others described financial rigidity or the lack of collaboration between institutions, organisations, and levels of care. In three of the innovation arenas, COPD patients received specialist services at home. Hence, they disrupted the established division of care, where local municipalities have operating responsibility for homecare services and regional healthcare trusts are responsible for specialist services. In one of these arenas, the hospital was responsible for the initiative during the project phase and covered its costs though project funding. Some healthcare providers worried that this funding arrangement was a barrier to adoption and spread. In Norway, home care is usually managed and funded by primary care services in the local municipalities, and the healthcare providers worried that during the everyday struggle over budgets and resources within the hospital, the management would not prioritise home care services or services not decreed by law. Several used the concept of ‘silo mentality’ or ‘silo organisation’ when referring to the division of labour and economic responsibility between primary and specialist care. One said:

It is always the same, money talks [...] The development is sad really, because it is not all about money; what about quality of life? For a chronically ill patient, cancer, COPD, or heart failure, you name it, just staying at home, that is great! I do acknowledge that it is an economic issue. The effect of the service must be demonstrated to justify the money spent. So, I guess it will depend on the results of the study [the informant refers to an ongoing study in which they measure number of days spent at the hospital and number of re-admissions to the hospital, and compare COPD patients receiving technology-supported PCC at home with COPD patients without PCC]. However, I reckon, for the hospital, the effect must be substantial to justify paying wages for all the involved professionals. To prove increased quality of life is not enough to keep the service up and running. (Informant 13)

While the healthcare providers described the involved organisations as capable and ready for handling projects, they were described as less ready and capable of paying for new technology-supported PCC practices within tight day-to-day budgets. The silo organisation between primary and secondary care was described as badly equipped for cross-institutional and cross-professional technology-supported PCC initiatives, particularly the complex interdependencies within and between departments and institutions related to who should pay for and who should benefit from new services.

### Domain 6 - the wider context

This domain relates to the simplicity, complicatedness, or complexity of the wider institutional and sociocultural context of the innovation. In our study, it became evident that the current policy strategies and funding models at local, regional, and national levels were simultaneously promoting and impeding the adoption and spread of the innovations. Across the four arenas, healthcare providers talked about the governmental promotion of e-health and PCC synchronously to uttering concerns about how local, regional, and national governing made secure funding of the innovations complicated.

In one arena, the previous mayor and city council had committed to the innovation and contributed financially to the initiative. Everything ran smoothly until a new mayor and a new city council were nominated. For this arena, a new local government meant that the expected funding and municipal support first became uncertain and then disappeared.

Another arena applied an explicit regional focus for its technology-supported PCC model. It consisted of a telemedicine centre staffed with highly trained COPD nurses delivering technology-supported PCC to patients in their homes, either in the local municipality or in one of the neighbouring municipalities. Here, some healthcare providers described municipal governing as complex and potentially interfering with successful adoption. The region consisted of numerous municipalities with relatively few COPD patients each and was therefore also in short supply of high-quality expertise on COPD care. Hence, this region was assessed as particularly suitable for high-quality COPD care at a distance. Nevertheless, during implementation, it became evident that for some small municipalities in this region, investing in homecare services was more than providing proper heath care for citizens; it also meant potential employment within the municipality. Paying a neighbouring municipality for remote COPD care was weighted against local employment in home care, and therefore also the numbers of taxpayers within the municipality. In this region, the advantages and disadvantages of technology support were considered in a wider municipal context. For small municipalities, the cost-benefit analysis for technology-supported PCC included a wider context than the healthcare services.

Some healthcare providers also talked about how national e-health politics, policy guidelines, and procurement projects enabled the adoption and spread of technology-supported innovations. Nordic governments are taking a pivotal role in technology development and implementation, aiming for national, standardised systems accessible to all healthcare organisations and institutions. Even if there were no national policies for technology-supported PCC at the time of our research, healthcare providers across the arenas talked about how e-health policies and politics could affect the adoption and spread of their innovations. In one arena, the informants said there was a rivalry between competing telemedicine services; therefore, the choice of vendor on the national governmental level could determine the future of the innovation.

As NASSS demonstrates, an organisation’s failure to move from a successful demonstration project to a fully mainstreamed service that is widely transferable and persists in the long term does not only relate to the work within the innovation arenas but also to the wider context of the innovations. However, contrary to NASSS, it is our interpretation that despite attitudinal, financial, and policy challenges at all the innovation arenas, the staff continues to promote the innovations.

### 7) The interaction between the domains.

While the six domains above can be distinguished analytically, the reality of any technology implementation project is that at an empirical level, the domains are inextricably interlinked and dynamically evolving. The seventh domain is about how much scope there is for adapting and co-evolving the technology and the service over time.

In our study, the healthcare providers across the arenas were eager to co-evolve the innovation. As one of the informants exclaimed, ‘There is no way around technology-supported PCC. It is here to stay!’ (Informant 14). This statement is representative of most of the healthcare providers in this study. At the same time, we have demonstrated that all the NASSS domains, except the first two, comprise complicated or complex conditions that could alter implementation. It is interesting to note that at present, only one of the four technology-supported PCC initiatives has been adopted into everyday practice, but in a modified manner, one is abandoned, two continue as innovation projects, and one of these has moved to a new location and healthcare trust. Still, it does not seem to affect the healthcare providers’ commitment to and belief in technology-supported PCC. On the contrary, it seems like the challenges with implementation and adoption have armoured their vison of PCC. To many, the current project and innovation arena are just one piece in a larger puzzle of care transformation for the future.

### Study strengths and limitations

This study is part of the 3P project (2016–2020) consisting of nine separate work packages. 3P has been practising ongoing network meetings with the project management team, the key healthcare providers, and the interdisciplinary researcher group throughout the project period, thus ensuring continuous dialogue and feedback on our preliminary findings. Familiarity with the context can be an asset and contribute to deeper discussions in the interviews as well as in the analysis. On the other hand, closeness to the field over time can also be a limitation; it might have affected the interviews, analysis, and interpretation of the results.

We have used the NASSS framework to analyse healthcare providers’ experiences with technology-supported patient-centred care initiatives. There are a few alternative frameworks that could have been used and that explicitly target staff perspectives, e.g., normalization process theory (NPT). We tested NPT in the early stages of the study and found it less intuitive translated to the interdisciplinary audience in the 3P project.

## Conclusion

In our experience, the seven NASSS domains are a feasible analytical framework for systematising, categorising, and comparing healthcare providers’ experiences with technology-supported PCC initiatives. The seven domains are comprehensive and easily translated to an interdisciplinary audience, and the framework is useful for throwing light on the levels of complexity and the main challenges for sustained adoption at each of the innovation arenas and for identifying key challenges for adoption and spread across the arenas. It is useful to generate a rich and situated narrative of the multiple influences on a complex programme; hence, the NASSS framework is useful for those seeking to plan, implement, and evaluate technology-supported healthcare programmes.

We believe that the NASSS framework is helpful in identifying and thereby dealing with potential problems early in the implementation process and to evaluate why a few initiatives succeed while others fail. Thus, it can contribute to making technology supported programmes sustainable and efficient solutions for the healthcare services of the future.

While the NASSS framework is useful for identifying and communicating challenges with the adoption and spread of the four technology-supported PCC initiatives, we find it less sensitive for capturing the dedication, enthusiasm, and passion for care transformation that we found among the healthcare providers in our study. It is our interpretation that when it comes to technology-supported PCC, the point of no return has passed for key healthcare providers. To them, technology-supported PCC is already a definite part of future healthcare services. How to overcome barriers and obstacles and implement them on a large scale is pragmatically approached. This study emphasises the need to look beyond the single project and increase knowledge about the healthcare providers and their visions as potential assets for care transformation, which might be critical for those seeking to plan, implement, and evaluate technology-supported health or social care programmes. We believe healthcare providers are essential for care transformation and for the development of new and superior ways of treating patients in the future.

## Data Availability

The data that support the findings are held by Stein Olav Skrøvseth, Director, Norwegian Centre for E-Health Research, but restrictions apply to their availability. The data were used under licence for the current study; thus, they are not publicly available. However, data can be obtained from the authors upon reasonable request and with permission from the Norwegian Centre for E-Health Research.
